# Localized Tenosynovial Giant Cell Tumor Originating From the Flexor Carpi Radialis Tendon Sheath: An Unusual Case

**DOI:** 10.7759/cureus.106867

**Published:** 2026-04-11

**Authors:** Lalit Panchal, Sagar Bagwe, Bhushan Rathod

**Affiliations:** 1 Orthopedics and Traumatology, Fortis S L Raheja Hospital, Mumbai, IND

**Keywords:** flexor carpi radialis, magnetic resonance imaging, radial artery, surgical excision, tenosynovial giant cell tumor, volar wrist swelling

## Abstract

Tenosynovial giant cell tumor (TGCT) is a benign synovial proliferative lesion most commonly involving the digits, with wrist involvement being relatively uncommon. Occurrence from the flexor carpi radialis (FCR) tendon sheath is particularly rare and may mimic a volar ganglion cyst clinically. We report a case of a 27-year-old male presenting with a gradually enlarging, painless swelling over the volar wrist. Magnetic resonance imaging demonstrated a well-defined lesion arising from the FCR tendon sheath in close proximity to the radial artery, without osseous involvement. Complete excision was performed, and histopathology confirmed localized TGCT. The patient had an uneventful recovery, with no recurrence at one-year follow-up. This report highlights the diagnostic challenge and emphasizes the importance of imaging and complete surgical excision.

## Introduction

Tenosynovial giant cell tumor (TGCT) represents a spectrum of benign proliferative lesions arising from synovium-lined structures, including tendon sheaths, joints, and bursae [1]. It is recognized as the second most common soft-tissue mass of the hand after ganglion cysts [2]. Although digital involvement predominates, lesions affecting the wrist are far less frequent, accounting for approximately 5%-15% of cases, while 65%-85% occur in the digits [3,4]. Involvement of the flexor carpi radialis (FCR) tendon sheath is exceedingly rare and sparsely reported in the literature. Based on growth pattern and extent, TGCT is classified into localized and diffuse variants, the former being more typical in the hand region. Volar wrist lesions pose particular diagnostic and surgical challenges due to their close anatomical relationship with the radial artery and flexor tendons. Because of overlapping clinical features, these masses are frequently misinterpreted as ganglion cysts or other benign soft-tissue tumors. We describe a rare case arising specifically from the FCR tendon sheath and discuss diagnostic considerations and management principles.

## Case presentation

**Figure 1:**
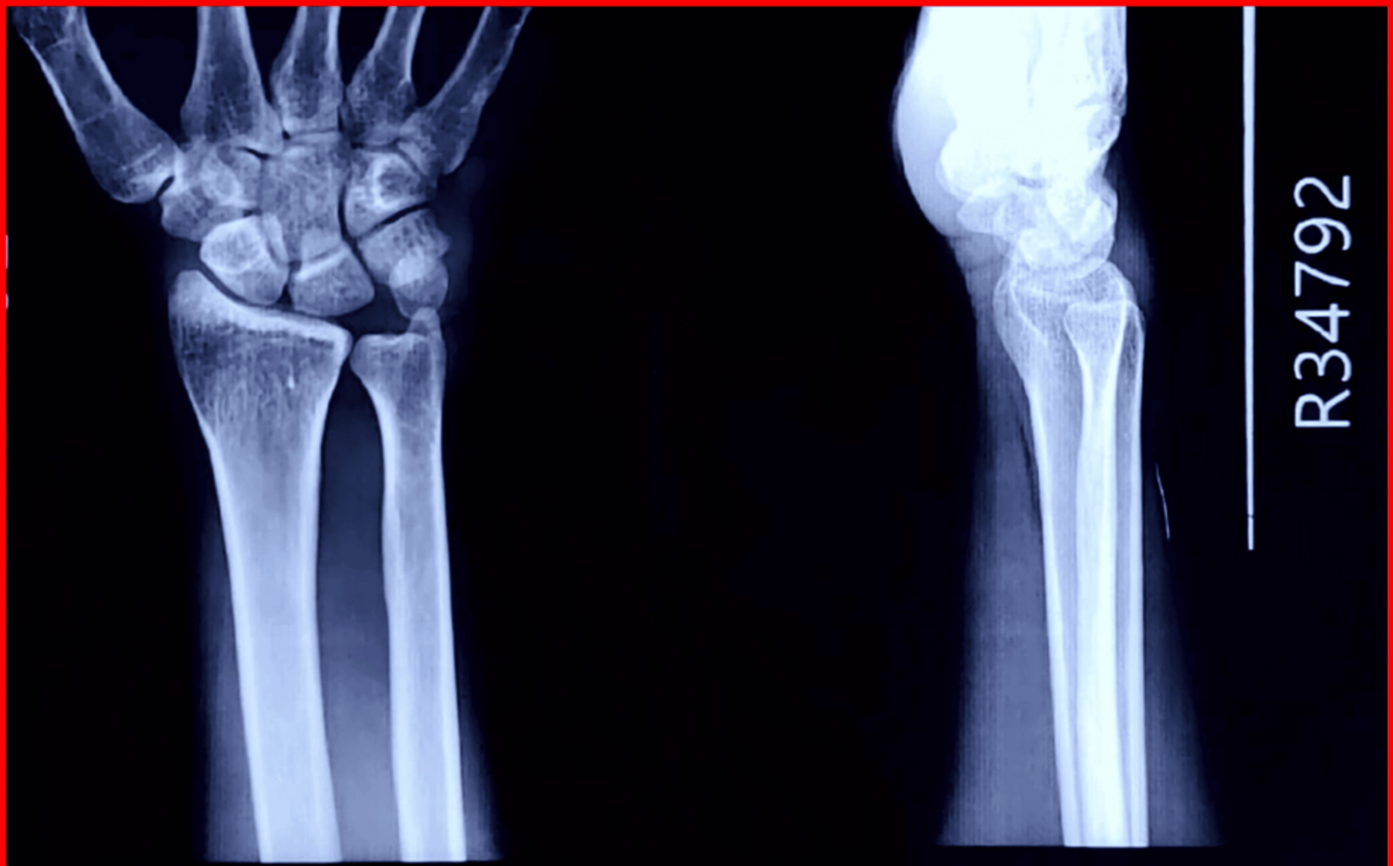
Anteroposterior radiograph of the wrist showing no bony abnormality

A 27-year-old male presented with a painless swelling over the volar aspect of the right wrist for six months. The swelling gradually increased in size without any preceding trauma or systemic illness. Mild discomfort during wrist flexion and thumb movements was noted, without sensory deficits. On examination, a firm, non-tender, well-defined mass measuring approximately 1 cm was present over the volar wrist. The swelling was mobile relative to the skin but moved with contraction of the FCR tendon. Plain radiographs showed no bony abnormalities (Figure 1).

**Figure 2:**
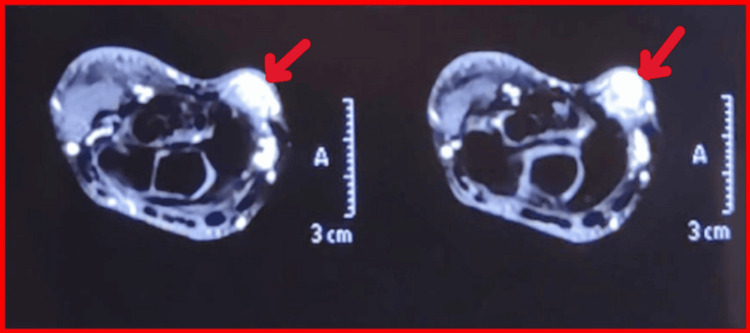
Axial T2-weighted MRI sections showing the lesion The TGCT is T2 hyperintense and is marked by a red arrow. TGCT: tenosynovial giant cell tumor; MRI: magnetic resonance imaging

**Figure 3:**
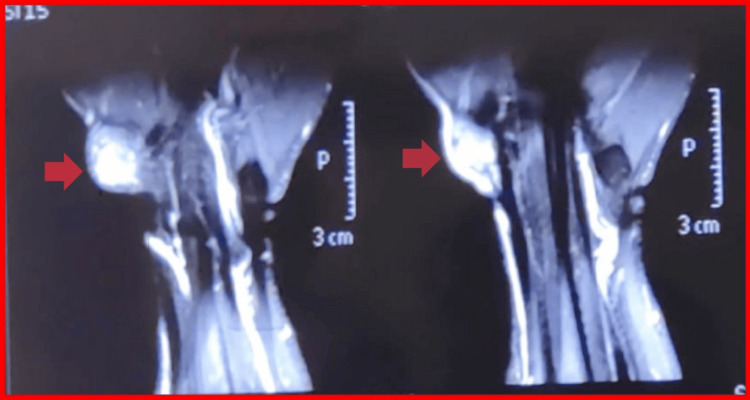
T2-weighted coronal images of the wrist The TGCT is hyperintense on this coronal MRI section, marked by a red arrowhead, with the radial artery appearing as a hyperintense vessel pushed laterally by the tumor. A blooming artifact due to hemosiderin deposits was also observed. TGCT: tenosynovial giant cell tumor; MRI: magnetic resonance imaging

**Figure 4:**
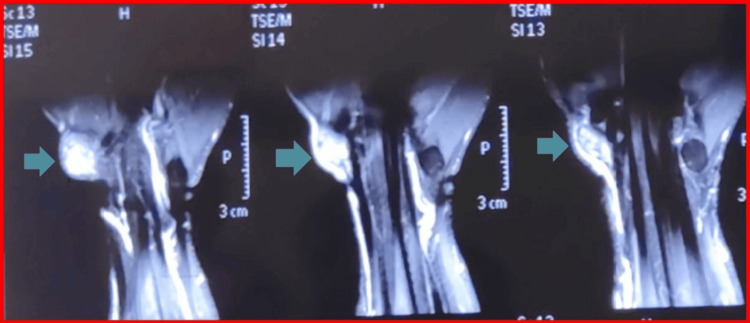
T2-weighted serial coronal MRI sections of the wrist These coronal MRI sections show the tumor displacing the radial artery laterally, both appearing as T2 hyperintense, with hypointense areas suggestive of hemosiderin deposits and blooming artifact on the MRI sections, and the FCR tendon appearing isointense. FCR: flexor carpi radialis; MRI: magnetic resonance imaging

Magnetic resonance imaging revealed a well-defined lesion arising from the FCR tendon sheath. The lesion demonstrated signal intensity similar to muscle on T1-weighted images and heterogeneous high signal on T2-weighted sequences, measuring 11 mm × 9 mm × 12 mm in dimensions. Gradient-echo sequences demonstrated blooming artifact consistent with hemosiderin deposition [5]. The radial artery was displaced laterally, without intra-articular extension (Figures 2, 3, 4). No bone marrow edema or intra-articular communication was detected.

**Figure 5:**
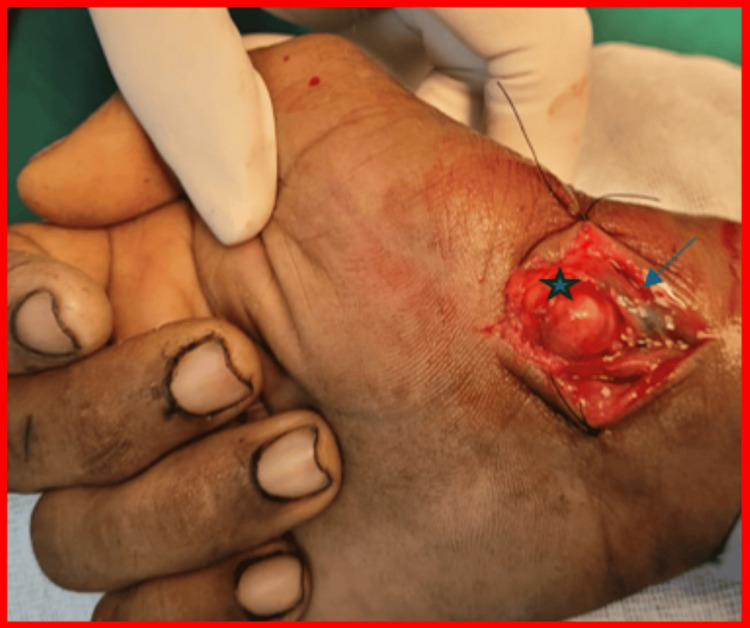
Intraoperative image showing the TGCT exposed The tumor mass arising from the FCR tendon is marked by a blue star, with lateral displacement of the radial artery (marked by a blue arrowhead) in this intraoperative image. TGCT: tenosynovial giant cell tumor; FCR: flexor carpi radialis

**Figure 6:**
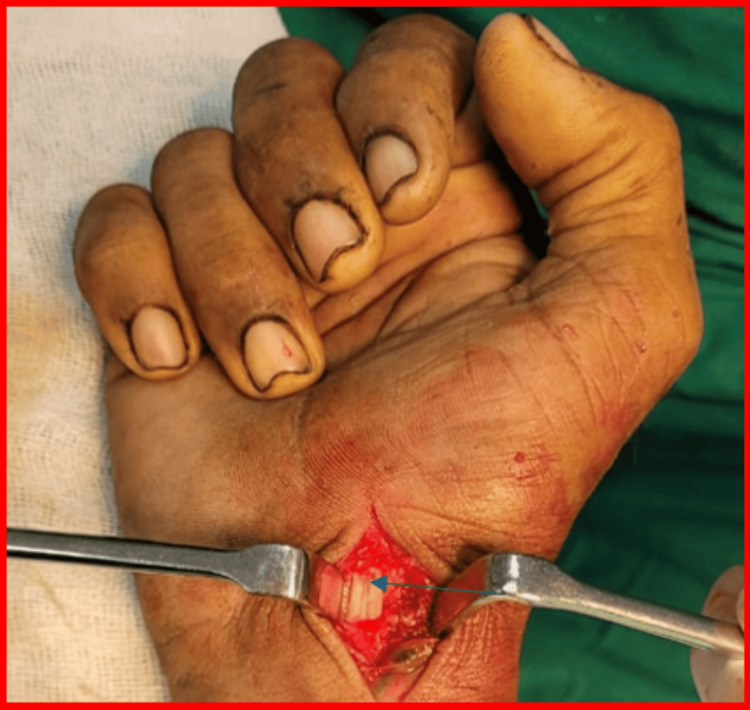
Intraoperative photograph after tumor excision The tumor bed is seen, with the FCR tendon visible and intact, marked by a blue arrow, and the radial artery lying lateral to the tendon. FCR: flexor carpi radialis

**Figure 7:**
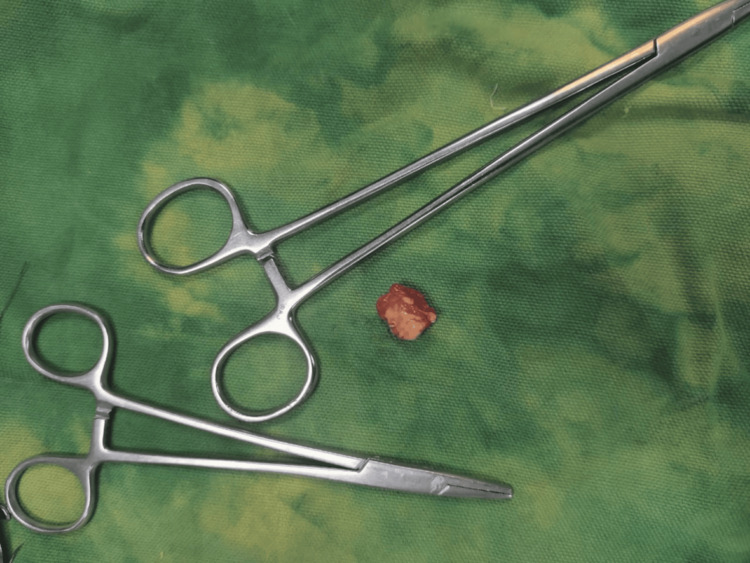
Intraoperative photograph showing the excised specimen Yellowish-gray, firm, spherical mass excised along with the synovial tissue from which it arose.

Surgical excision was performed using a volar approach under regional anesthesia. The lesion was identified as arising from the FCR tendon sheath and was excised en masse along with a portion of the sheath, while preserving tendon integrity. The radial artery was carefully protected (Figures 5, 6, 7).

**Figure 8:**
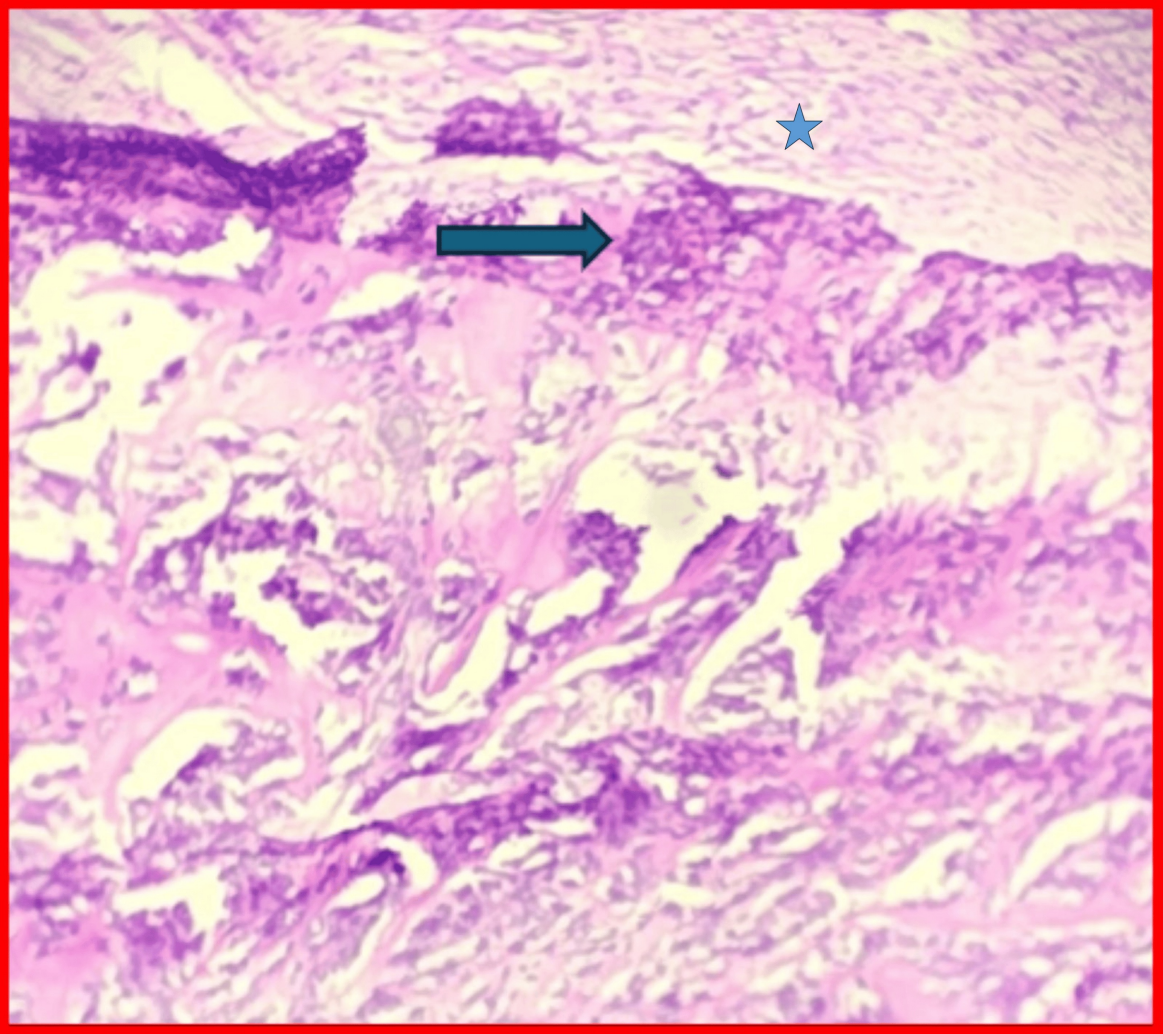
Histopathological sections showing multinucleated giant cells, mononuclear stromal cells, and hemosiderin deposition (H&E stain) The blue arrow shows multinucleated giant cells characteristic of a TGCT, and the blue star indicates the stroma with mononuclear clear cells. The atypical T2 hyperintensity correlates with heterogeneous histopathology, where regions with reduced hemosiderin deposition and increased cellularity demonstrate higher T2 signal intensity, while areas rich in hemosiderin show low signal and blooming. H&E: hematoxylin and eosin; TGCT: tenosynovial giant cell tumor

Histopathological examination demonstrated mononuclear cells, osteoclast-like multinucleated giant cells, and hemosiderin deposition, confirming localized TGCT (Figure 8). The postoperative period was uneventful, and no recurrence was observed at one-year follow-up. However, recurrence rates range from 4%-30%, most commonly within the first two to three years [6,7]. The atypical T2 hyperintensity correlates with heterogeneous histopathology, where regions with reduced hemosiderin deposition and increased cellularity demonstrate higher T2 signal intensity, while areas rich in hemosiderin show low signal and blooming.

## Discussion

Wrist involvement of TGCT is uncommon, and lesions arising from the FCR tendon sheath are rare. These lesions are often misdiagnosed as ganglion cysts due to a similar presentation [2].

Magnetic resonance imaging plays a crucial role in diagnosis and preoperative planning. Characteristic findings include well-defined lesions with variable signal intensity influenced by hemosiderin content. Areas of low signal correspond to dense hemosiderin deposition, while relatively high T2 signal reflects reduced hemosiderin and increased stromal cellularity [5].

Complete excision, including the involved tendon sheath, is the treatment of choice and reduces recurrence rates [6,7]. Careful dissection is required due to proximity to structures such as the radial artery. The differential diagnosis includes ganglion cyst, fibroma of the tendon sheath, and other soft-tissue tumors; however, blooming on GRE sequences and a solid nature favor TGCT.

TGCT is a benign lesion with potential for local recurrence, particularly in cases of incomplete excision [8,9]. The localized form typically presents as a slow-growing, painless mass.

## Conclusions

TGCT arising from the FCR tendon sheath represents a rare clinical entity that can closely mimic more common conditions such as volar wrist ganglion cysts. This case underscores the importance of maintaining a high index of suspicion when evaluating atypical volar wrist swellings, particularly those that are firm, slowly progressive, and associated with tendon movement. Magnetic resonance imaging plays a pivotal role in preoperative assessment by accurately delineating lesion extent and its relationship to adjacent critical structures, especially the radial artery. Such detailed imaging is essential for safe surgical planning.

Complete surgical excision, including removal of the involved tendon sheath, remains the cornerstone of management and is crucial in minimizing recurrence risk. Meticulous dissection, with preservation of surrounding neurovascular structures, ensures optimal functional outcomes. Early diagnosis and appropriate intervention can lead to excellent clinical recovery with low recurrence rates. This case highlights the need for careful clinical evaluation, appropriate imaging, and precise surgical technique in managing rare presentations of TGCT in the wrist. A limitation of this report is the relatively short follow-up duration; long-term follow-up is essential for documentation of recurrence.
